# Using AI-Based Gait Analysis to Establish a 5-Meter Walk Time Cutoff for Discriminating Alzheimer's Disease

**DOI:** 10.7759/cureus.95491

**Published:** 2025-10-27

**Authors:** Tadatoshi Inoue, Shogo Sawamura, Takashi Nagai, Kengo Kohiyama, Takahiro Takenaka, Tatsuya Sera, Lisa Senba

**Affiliations:** 1 Department of Rehabilitation, Heisei College of Health Sciences, Gifu, JPN; 2 Faculty of Health Sciences, Kumamoto Health Science University, Kumamoto, JPN

**Keywords:** alzheimer's dementia, artificial intelligence, cutoff value, gait analysis, older adults

## Abstract

Introduction

A decline in gait function has been reported to occur early in Alzheimer's disease (AD), a major cause of dementia, suggesting that gait analysis may be a useful tool for dementia screening. However, a simple and practical analysis method or clear cutoff values have yet to be established. This study aimed to analyze gait function using the AI-powered smartphone application "Toruto," identify gait indicators characteristic of elderly patients with AD, and propose effective cutoff values for dementia discrimination.

Methods

A total of 147 participants were included in the study: 86 healthy elderly individuals and 61 elderly patients with AD (102 female patients and 45 male patients). Exclusion criteria included the use of a cane, the presence of pain during walking, or the need for walking assistance. Gait function at a normal walking speed was analyzed by the AI of "Toruto" to assess speed, rhythm, and left-right asymmetry. An unpaired t-test was used to compare the two groups, and cutoff values for dementia discrimination were calculated using receiver operating characteristic (ROC) curve analysis. The significance level was set at p < 0.05.

Results

The AD group showed a significant decline in gait speed (12.12 versus 3.98 sec/5 m), rhythm (10.92 versus 6.51), and left-right asymmetry (6.34 versus 2.15) compared with the healthy control group (p < 0.05). ROC curve analysis revealed that using a gait speed cutoff value of 5.85 sec/5 m yielded a sensitivity of 96.7% and a specificity of 96.5%. A rhythm cutoff of 7.06 (sensitivity: 80.3%, specificity: 65.1%) and a left-right asymmetry cutoff of 2.25 (sensitivity: 75.4%, specificity: 67.4%) were also effective discriminative indicators.

Conclusion

Gait analysis using the AI-powered smartphone application "Toruto" is effective for distinguishing Alzheimer's disease. This study demonstrated that cutoff values, such as 5.85 seconds for a 5-meter walk, serve as practical screening indicators for dementia. As an objective biomarker reflecting cognitive decline, gait function is expected to be useful for the early detection of Alzheimer's disease in both community and clinical settings.

## Introduction

As of 2024, Japan's aging rate has reached 29.3% [1]. To sustain a healthy social security system, preventing individuals from becoming dependent on care and extending healthy life expectancy are being promoted [2]. Common causes of needing care include dementia, fractures, and falls [3]. According to a survey by the Ministry of Health, Labour and Welfare, Alzheimer's disease (AD) is the most common cause of dementia [4].

Kim et al. reported that gait function declines from an early stage in patients with Alzheimer's disease [5]. Similarly, Nadkarni et al. reported that patients with early-stage Alzheimer's disease exhibit a slower gait speed [6], suggesting that detailed gait analysis could be an indicator for the early detection of Alzheimer's disease [7].

Conventional methods for gait analysis often use pressure gauges, which are expensive and require trained professionals to operate. Furthermore, although the relationship between cognitive and gait function is known, a clear cutoff value has not yet been established.

This study aimed to identify gait parameters using the AI-powered smartphone application "Toruto" that can distinguish individuals with Alzheimer's disease from healthy older adults and to establish optimal cutoff values for screening.

## Materials and methods

Subjects

The subjects for this study were 86 community-dwelling older adults participating in a health club and 61 individuals with Alzheimer's disease who use day-care services. Recruitment for the health club participants was conducted through the city's website, posters at city halls and community centers, and guidance from local comprehensive support center staff and life support coordinators after health checkups. The principal investigator and project manager explained the purpose of the study to the participants, and those who provided consent were included. As the measurements involved gait, the inclusion criteria for the subjects were as follows: health club participants had to not have received long-term care certification, while day-care users had to be 75 years or older with a diagnosis of Alzheimer's disease. Exclusion criteria included using assistive devices such as canes for mobility, experiencing pain during walking, or requiring assistance with walking. The final cohort consisted of 147 subjects (102 female patients and 45 male patients). Subject demographics are presented in Table 1 and Table 2.

**Table 1:** Attributes of the subjects (N = 147) Regularity (standard deviation): regularity measures the position of the left and right ankle joints and is the standard deviation of the time taken during the swing phase to install per step. Asymmetry (left/right ratio): the left-right difference is the ratio of the left-right difference when the time of the left-right stance period is added and the ratio of the left-right difference is 100. MMSE: Mini-Mental State Examination

Attribute	Minimum value	Maximum value	Mean	Standard deviation
Age	75	98	85.84	4.80
MMSE	5	23	17.92	4.90
Speed (sec/5 m)	2.2	60	7.36	7.41
Regularity (standard deviation)	3.1	20	8.34	4.29
Asymmetry (left/right ratio)	20	67	50.81	6.30

**Table 2:** Care level distribution for patients with Alzheimer's disease (n = 61)

Care level	Number (%)
1	37 (60.7%)
2	21 (34.4%)
3	2 (3.3%)
4	1 (1.6%)
5	0 (0%)
Total	61 (100%)

Measurement items

We evaluated age and gait function in a group of healthy older adults participating in a community health club (healthy group). In a separate group of patients with Alzheimer's disease attending a day service center (AD group), we evaluated age, gait function, cognitive function, and care level. Cognitive function was assessed using the Mini-Mental State Examination (MMSE), a widely used 30-point screening tool. A score below 24 on the MMSE is considered indicative of cognitive impairment [8]. The participants' care levels were officially certified by local municipal governments under Japan's public Long-Term Care Insurance (LTCI) system. This system categorizes individuals based on their physical and mental needs into stages of independence, support (Yo-shien levels 1-2), or care (Yo-kaigo levels 1-5) [9]. The MMSE and care level were administered exclusively to the Alzheimer's group, as these assessments were deemed less relevant to the healthy control participants.

Gait function was assessed using the smartphone application "Toruto" (Exa Home Care Inc., Tokyo, Japan). Gait was assessed during a single trial of walking at a self-selected, comfortable speed. The reliability and validity of this application have been established in previous studies [10]. The application utilizes a proprietary algorithm developed by ExaWizards Inc., which is based on the OpenPose machine learning library for posture estimation and environmental detection. It extracts the skeletal structure and analyzes a 5-meter walking segment by setting landmarks at the center of the neck and the left and right hip joints [11]. Three gait parameters were obtained from the analysis: speed, regularity, and asymmetry. Speed was calculated based on the individual's body size. Regularity was determined from the standard deviation of the swing phase time, calculated from the positions of the left and right ankle joints. Asymmetry was calculated from the time ratio of the left and right stance phases [12,13]. For the measurement, an iPad was positioned at a height of 150 cm from the floor and 1 meter behind the starting line. Participants walked a total distance of 10 meters, and the central 5-meter segment was used for the analysis.

An unpaired t-test was used for the two-group comparison between the healthy group and the AD group. To determine the optimal cutoff values of the gait parameters for identifying cognitive impairment, a receiver operating characteristic (ROC) curve analysis was performed. The cutoff values were determined using the Youden index. The area under the ROC curve (AUC), sensitivity, and specificity were calculated.

A p-value of less than 0.05 was considered statistically significant. All data were presented as mean ± standard deviation (SD). Statistical analyses were performed using SPSS version 24 (IBM Corp., Armonk, NY).

## Results

In this study, we evaluated the gait function of elderly individuals aged 75 years and older to find an indicator for distinguishing cognitive function. The healthy group had a mean age of 85.65 years, with an average gait speed of 3.98 sec/5 m, a regularity score of 6.51, and a left-right asymmetry score of 2.15. The Alzheimer's group had a mean age of 86.10 years, an average MMSE score of 17.92, an average long-term care level of 1.46, a gait speed of 12.12 sec/5 m, a regularity score of 10.92, and a left-right asymmetry score of 6.34. Compared to the healthy group, the Alzheimer's group showed a slower gait speed, decreased and more irregular rhythm, and increased variability, indicating unstable gait (Table 3).

**Table 3:** Two-group comparison of gait function (N = 147) Values are presented as mean ± SD. Statistical analysis was performed using an unpaired t-test. *p < 0.05, **p < 0.01 MMSE: Mini-Mental State Examination, SD: standard deviation

Attribute	Alzheimer group (n = 61)	Healthy group (n = 86)	t-value	p-value	Effect size (d)
Age	86.10 ± 5.92	85.65 ± 3.85	0.517	0.606	0.09
MMSE	17.92 ± 4.90	-	-	-	-
Speed (sec/5 m)	12.12 ± 9.62	3.98 ± 1.06	6.581	<0.01	1.30
Regularity (SD)	10.92 ± 4.92	6.51 ± 2.52	6.423	<0.01	1.19
Asymmetry (left/right ratio)	6.34 ± 6.30	2.15 ± 2.22	2.709	<0.01	1.02

Gait function was found to be an effective indicator for dementia screening, with the following cutoff values: a speed of 5.85 sec/5 m (sensitivity: 96.7%, specificity: 96.5%), a regularity score of 7.06 (sensitivity: 80.3%, specificity: 65.1%), and a left-right asymmetry score of 2.25 (sensitivity: 75.4%, specificity: 67.4%) (Table 4).

**Table 4:** Cutoff scores for each item (N = 147) **p < 0.01 AUC: area under the curve, CI: confidence interval

Attribute	Cutoff value	AUC	Sensitivity	Specificity	95% CI	p-value
Speed (sec/5 m)	5.85	0.984	0.967	0.965	0.964-1.000	<0.01
Regularity (standard deviation)	7.06	0.784	0.803	0.651	0.709-0.858	<0.01
Asymmetry (left/right ratio)	2.25	0.761	0.754	0.674	0.679-0.842	<0.01

## Discussion

This study demonstrated that gait function is not merely a physical ability but serves as a highly useful biomarker reflecting the state of cognitive function. Among individuals aged 75 years and older, three gait parameters (speed, rhythm, and laterality) were effective in discriminating Alzheimer's disease (AD). Notably, a highly practical cutoff value of 5.85 seconds for a 5-meter walk yielded excellent sensitivity and specificity.

Conventional screening tools such as the Mini-Mental State Examination (MMSE) and the Montreal Cognitive Assessment (MoCA) require professional administration and are time-consuming. In contrast, gait measurement is brief and non-invasive, and capable of objectively capturing subtle changes in cognitive function [14]. In particular, the 5-meter walk threshold of 5.85 seconds enables immediate evaluation in community-based integrated care settings and demonstrates the potential for implementation as a preclinical dementia risk screening tool via smartphone-based self-assessment.

Simultaneous decline in gait and cognitive function has been reported to predict future dementia onset [15,16], and our findings provide specific and feasible indicators for introducing first-stage community screening. The observed slowing of gait speed may be attributed to dysfunction of the frontal-executive network. Age-related and AD-related declines in executive function and attentional resources have been shown to impair gait control and contribute to gait slowing [17,18]. Furthermore, accumulation of amyloid-β and tau proteins in AD has been reported to disrupt neuronal transmission efficiency between cortical and subcortical circuits [19].

Irregular gait rhythm and laterality may reflect asymmetric neurodegeneration in AD, which often progresses unevenly from the parietal to the temporal lobes and basal forebrain. Previous studies have suggested that such asymmetry can serve as an auxiliary marker for disease discrimination [20]. This asymmetrical progression may affect arm swing and lower limb balance, manifesting as gait laterality. In addition, early pyramidal tract involvement has been described in AD [21], which may further explain the observed differences in gait speed, rhythm, and symmetry between AD and cognitively healthy groups.

As demonstrated in this study, smartphone-based gait analysis represents a promising tool for primary dementia prevention, particularly in communities with limited medical and caregiving resources. Continuous longitudinal accumulation of individual gait data could facilitate the development of early predictive models for cognitive decline and provide a foundation for personalized interventions.

Limitations and future directions

This study has several limitations. Although we excluded subjects with lower limb pain, those undergoing treatment, and those who use canes, gait function is influenced by many factors, including lower limb muscle strength, balance, joint range of motion, vision, and somatosensory perception. Therefore, it is difficult to completely distinguish whether the observed decreases in speed, regularity, and left-right asymmetry are specific to Alzheimer's disease or are caused by other physical factors, such as sarcopenia or age-related changes. In the future, a more comprehensive evaluation of physical function using muscle strength assessments, balance tests, and activity measurements will be needed to more clearly investigate the relationship between gait decline and dementia.

The cutoff values presented in this study are based on our specific study population, and their applicability to other populations has not yet been verified. To confirm external validity, large-scale, multicenter studies will be necessary.

## Conclusions

This study demonstrated that gait analysis using the smartphone application "Toruto" can effectively distinguish Alzheimer's disease (AD) from normal aging among community-dwelling older adults. The AD group showed slower gait speed, greater rhythm irregularity, and increased laterality, with a 5-meter walk cutoff of 5.85 seconds showing high sensitivity and specificity.

The major strength of this study is its demonstration of a simple, non-invasive, and practical screening method that does not require specialized equipment or professional assessment. By capturing gait characteristics that reflect cognitive decline in daily life, this approach enables objective and rapid evaluation in community and home-care settings. These findings suggest that smartphone-based gait analysis may serve as a feasible tool for early dementia detection and could support future predictive and personalized intervention models.
